# Navigating a varying reward environment in childhood and adolescence

**DOI:** 10.1038/s41598-025-05725-3

**Published:** 2025-07-02

**Authors:** Louise Neil, Vincent Valton, Essi Viding, Diana Armbruster-Genc, Vivien Vuong, Katy Packer, Molly Sharp, Jonathan P. Roiser, Eamon McCrory

**Affiliations:** 1 Division of Psychology and Language Sciences, University College London, 26 Bedford Way, London, WC1H 0AP UK; 2 Institute of Cognitive Neuroscience, University College London, London, UK; 3 Anna Freud, London, UK

**Keywords:** Computational modelling, Learning rates, Reinforcement learning, Child development, Belief updating, Psychology, Risk factors

## Abstract

Optimal reward learning requires individuals to adjust their learning rates – the extent to which new information replaces old. Learning rates should be higher in volatile environments, where new information is more salient, and lower in stable environments, where the longer-term history of outcomes is more predictive. It is not known, however, whether this adjustment in learning rates changes with age or is associated with better mental health and social functioning. We administered a child-friendly probabilistic reinforcement learning task with both fixed and fluctuating reward schedules to 121 participants aged 8–16 years. Adjustment of learning rates across childhood and adolescence to suit the levels of uncertainty in the environment did not differ by age, nor was it associated with better mental health and social functioning. Instead we found that learning rates for worse-than-expected outcomes generally decreased with age, temperature increased with age and higher learning rates, specifically during positive stable environments, were associated with greater self-reported prosocial behaviour. Our results highlight the exaggerated impact of negative feedback on children and suggest an increase in exploratory behaviour between childhood and adolescence.

## Introduction

Navigating complex social environments requires the capacity to associate stimuli and actions with rewards and to update these associations appropriately when contingencies change. Advances in computational modelling mean that the identification of specific information processing components driving developmental changes in associative learning is now possible. One parameter of particular interest is learning rate: the extent to which differences in expected and actual outcomes are weighted when beliefs are updated. It is still unclear, however, whether learning rates change across development and if so, how. Several studies have identified age-related decreases in learning rates following negative feedback^1–3^, however, the picture is not entirely consistent, with other researchers finding no age-related changes^4,5^, or even the opposite trajectory whereby learning rates increase with age^6^. In light of these different patterns of findings, it was recently proposed that rather than the ‘settings’ of learning rates changing with development, individuals may improve at contextualising prediction errors within the environment in which they occur, and weighting them accordingly^1,7^.

One contextual factor crucial to the salience of prediction errors is the level of uncertainty in the environment. The extent to which the updating of contingency estimates is adjusted in situations of varying volatility has been termed ‘second-order’ reward learning^8^. In a stable environment, the learning rate should ideally be low, because in this context the history of prior outcomes is more predictive of future contingencies. In a volatile environment, however, new information is more salient and therefore learning rates should be higher^9^. The capacity to track the statistics of the reward environment and incorporate changes in environmental volatility when updating reward contingencies is therefore essential to effective reward learning, but we do not know the extent to which this ability changes with age.

Over the past decade it has become increasingly clear that atypical reinforcement learning is a feature of mental health disorders^10–12^ although the mechanisms linking atypical reinforcement learning to poor mental health outcomes are poorly delineated. One possibility is that over- or under-weighting the salience of new information may compromise effective decision making, potentially resulting in missed opportunities or increased vulnerability to stressors (see^13^ for a review). There is some evidence that individuals with mood and anxiety disorders have lower learning rates following positive outcomes and higher learning rates following negative outcomes^14^,whereas anxiety has been associated with lower flexibility in learning rates when reward contingencies change^15,16^. While there is some understanding of how learning rates relate to psychiatric symptomatology in adulthood, the relationship between value updating and the emergence of mental health symptoms in childhood remains unclear. It is plausible that children who can adapt their learning rates to different contexts, for example by incorporating the volatility of the reward environment when weighting new information, are better able to negotiate new learning environments. This may be especially critical as children move from childhood to adolescence, when both educational tasks and social interactions become increasingly complex and have to be negotiated more independently – yet we have limited data regarding how volatility and valence interact to influence learning rates in children of different ages.

Another aspect of decision making relevant to maximising returns from the environment is the extent to which individuals explore new options versus sticking to safe, familiar options which have been rewarded in the past. In computational models this behaviour is indexed by a ‘temperature’ parameter, where higher scores reflect greater exploration and lower scores reflect exploitation of a high value option. There is much evidence that exploratory behaviour decreases from childhood to adulthood^1,2,17,18^; although see^3,19^ implying that as individuals grow older they select stimuli known to be associated with rewards more frequently. Higher temperature reflects noisier decision making and therefore, perhaps for this reason, it has been positively associated with symptoms such as anhedonia^20^, however a recent simulated meta-analysis found no difference in inverse temperature between anxiety/depression patients and a control comparison group^14^. As yet, little is known about the nature of the relationship between temperature and mental health/social functioning in children and young adolescents. This may be of particular relevance given the centrality of exploration to social and emotional learning in youth.

In the current study we investigated whether learning rates in stable and volatile conditions, and following positive and negative outcomes, changed with age. We cover the early adolescent period^21^ a time of significant social and emotional changes. We hypothesised that there would be age-related increases in children’s adjustment of learning rates to match the volatility of the environment. We also hypothesised that greater adjustment of learning rates in response to the changing volatility of a given environment would be associated with better mental health and social functioning. Given considerable evidence that exploratory behaviour decreases with age^7^, it was hypothesised that there would be age-related decreases in the temperature parameter.

## Methods

### Participants

Two-hundred and twenty-nine participants (141 male) aged from 8 to 16 years (mean = 11.79, SD = 2.33),recruited through state-funded, non-fee-paying primary and secondary schools in Greater London and the South East completed a child-friendly probabilistic learning task featuring stable and volatile conditions. All participants scored > 70 on the two subtest version of the Second Wechsler Abbreviated Scales of Intelligence (WASI-2)^22^, mean = 99.07, SD = 13.57). Consistent with the procedure adopted by^9,23^ only data from children completing order 1 of the counterbalanced task (stable condition followed by volatile condition, n = 121) were included in primary analysis. These participants ranged in age from 8 to 16 (mean age = 11.69, SD = 2.40; WASI mean = 99.50, SD = 12.78). The remaining 108 participants who were counterbalanced to order 2 (volatile condition then stable condition) were included in additional analysis (Supplementary Information). A subset of n = 67 children from order 1 aged 11 and above completed a self-report mental health questionnaire. This study was carried out in accordance with the Declaration of Helsinki. The procedures used were approved by the University Research Ethics Committee. In order to ensure as representative a sample as possible, an opt-out consent procedure was used whereby all parents were informed two weeks before the school visit that their child, along with the rest of their class, would be participating in the research project, and were invited to opt their child out via the school or directly to the research team if they did not want them to take part. All children gave their informed, written assent to participate.

### Measures

#### Experimental task

**Fig. 1:**
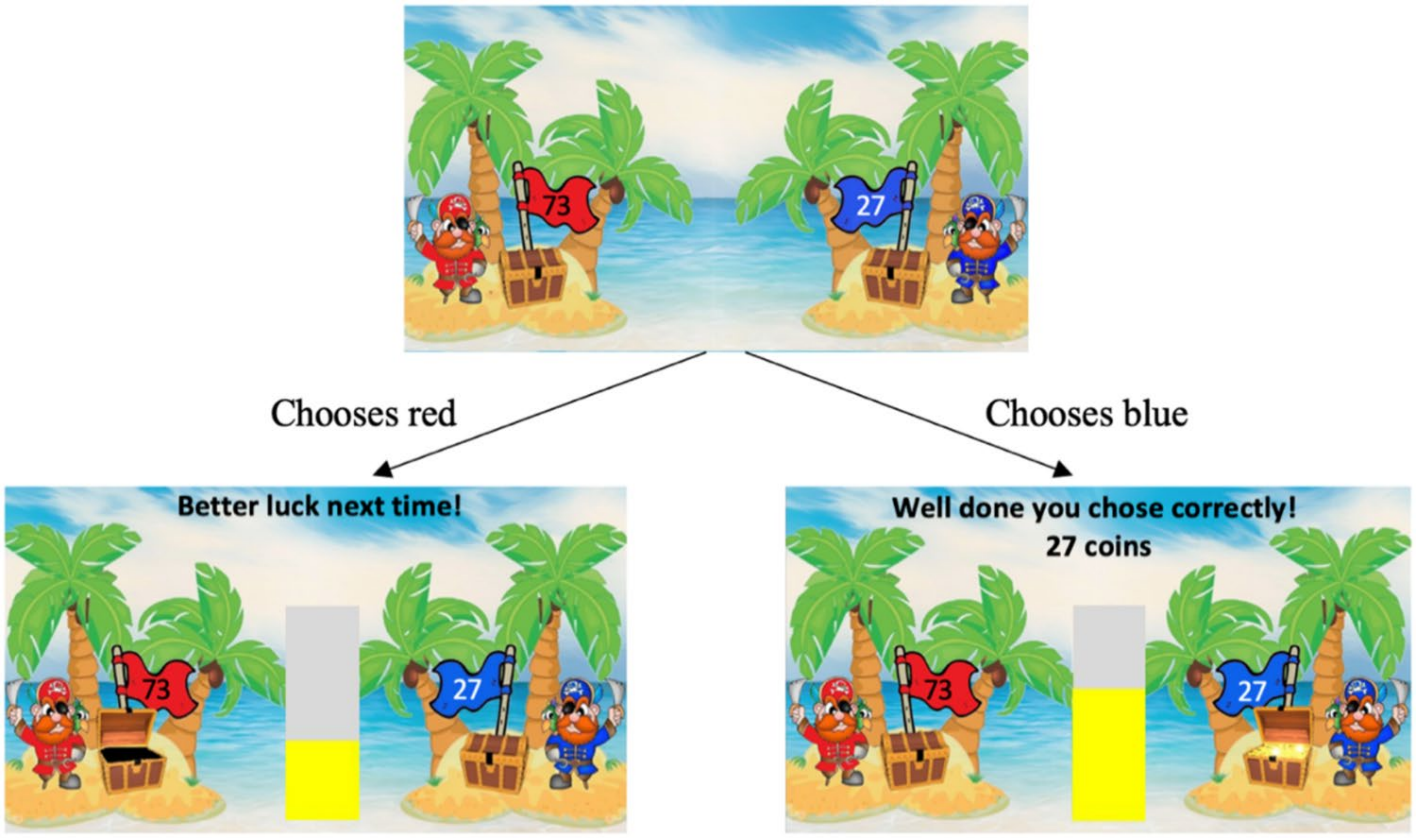
Reward learning task. Children were asked to choose which pirate’s chest they thought had the gold coins (indicated with a key press) before the rewarded stimulus was revealed. In this example trial, if a participant chooses the red pirate, an empty chest is revealed, and no points are awarded. If a participant chooses the blue pirate, a treasure chest is revealed, and the number of points indicated on the flag (27) is awarded.

We used a reinforcement learning paradigm in which volatility is manipulated, originally introduced by^9^ and adapted to a child-friendly version with a pirate theme by^23^. Participants were shown an image of two pirates, each standing next to a treasure chest with a flag in it (Fig. 1). The treasure chests each displayed randomly selected reward values ranging from 0 to 100, always with a combined value of 100. Participants were told that only one chest contained the number of gold coins indicated by the flag (the rewarded stimulus), and that the other was empty, regardless of the number indicated on the flag (the non-rewarded stimulus).

Participants initially watched 20 trials in which two pirates (yellow and green) opened their chests to reveal which one contained gold coins and which was empty. The ratio of outcomes for the more frequently reward pirate’s chest over the other was set at 80:20. Before watching, participants were told that the chest containing the gold coins would not always be the same one. Afterwards, to check children’s understanding of the task, participants were asked which chest contained the treasure most often, and to indicate the perceived ratio by choosing a point on a scale ranging from ‘all yellow’ to ‘all green’. If a participant could identify the ‘highest-probability’ pirate, by pointing within the correct half of the scale, they went on to complete the main task. The familiarisation stage was repeated a maximum of one time, for any child unable to identify the correct pirate the first time.

During the main task, participants were told that they were playing for a real reward (vouchers worth up to £10). Participants were again presented with two pirate’s chests (one red, one blue) but had to choose which pirate they thought had the gold coins (indicated with a key press) before the rewarded stimulus was revealed, in order to win points. The split of 100 points was random and therefore the number of points revealed by the ‘winning’ pirate on each trial was also random. The probability of winning points was not connected to point value. Participants were not told anything about the reward structure, only that to get to the highest level they should try and win gold coins every time. The task comprised 160 trials divided into two conditions: in the stable condition, the ratio of the red pirate being rewarded remained at 75:25 for all 80 trials. In the volatile condition, the ratio changed from 80:20 to 20:80 every 20 trials. All 160 trials were administered in a single session but the order of conditions (stable or volatile first) was counterbalanced across participants.

During the task, whenever a child chose the rewarded stimulus their points total, represented by a bar in the centre of the screen, increased in accordance with the value displayed on the chosen pirate’s flag. This was accompanied by additional visual “Well done, you chose correctly” and auditory (the sound of coins dropping) feedback. When participants chose the non-rewarded stimulus they were given the visual feedback “Better luck next time”, and the bar chart level remained the same. Whenever the bar was filled, participants were shown a screen informing them they had reached a new level, before the game continued. At the end of the game participants told their final level and points total e.g., “You reached level 10 and collected 1500 points”. All participants were told they had achieved enough points to receive a real, monetary reward.

#### Modelling of learning rates

The learning rates used to address the study aims and hypotheses were generated via a computational modelling approach. This approach was used to analyse participants’ decisions during the task on a trial-by-trial basis. Participants’ data was fit to a series of twelve reinforcement learning models with increasing complexity (Supplementary Fig. S1). We started testing simple assumptions, such as whether participants performed randomly (Null model), simply updated behaviour based on immediate feedback (“win-stay/lose-shift”), and iteratively improved the models until we could capture the participants’ decision patterns as accurately and parsimoniously as possible. All models were implemented using hierarchical Bayesian estimation in Stan^24^, allowing us to recover parameter estimates accurately^25^, with priors specified across all participants. Generatively, this modelling approach describes that participants are expected to come from a common group-level distribution, such that participants’ parameters are then expected to be similar to one another. The Widely Applicable Information Criterion (WAIC) scale and K-fold cross validation (K-fold CV) were used to compare model fits^26,27^. We defined differences in model evidence (Δ5-fold CV) as: weak (0–2); positive (2–6); strong (6–10); and very strong (> 10)^28^. Please see Supplementary Information for a fuller and more detailed description of model testing and fit.

**Fig. 2:**
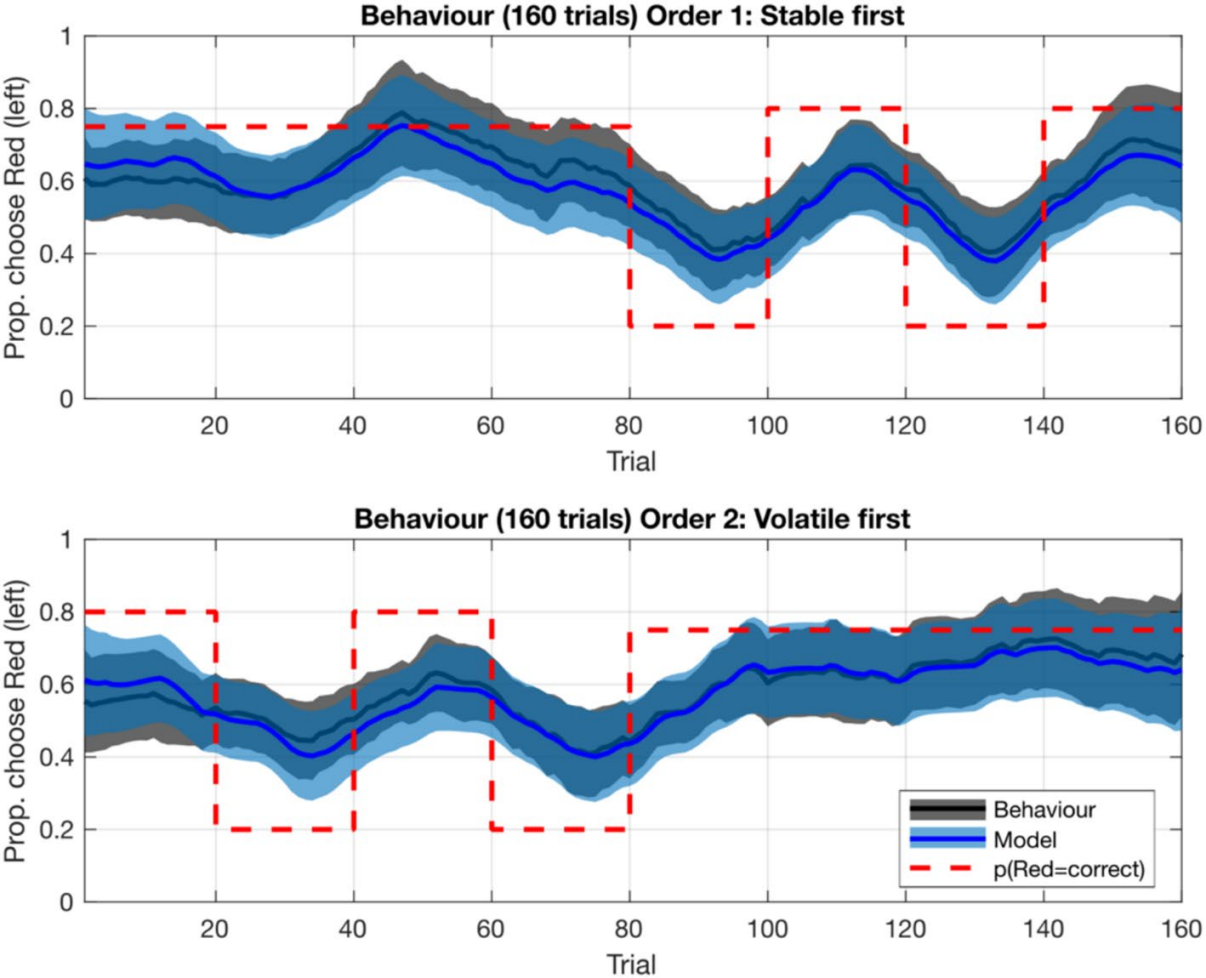
Mean moving average performance throughout the course of the task, by task order. The figures show the proportion of times participants chose the red pirate, throughout the task. ‘Model’ indicates the simulated choices on each trial from the winning model (i.e., M11: Temperature, Positive Stable LR, Negative Stable LR, Positive Volatile Learning Rate, Negative Volatile LR, with the blue line and light blue shading indicating the mean and 95% highest density interval (HDI) of simulated choices, respectively. ‘Behaviour’ indicates the empirical data from participants, with black line and grey shading indicating the mean and 95% HDI of actual choices, respectively. The simulated choices for each trial were created using each participant’s individual set of parameters, and then averaged across participants on each trial in the same way as the empirical data.

The model with the best fit (Supplementary Fig. S1) included four learning rate parameters: stable condition for positive prediction errors (i.e. better than expected outcome), stable condition for negative prediction errors (i.e. worse than expected outcome), volatile condition for positive prediction errors, volatile condition for negative prediction errors; and a temperature parameter. Figure 2 shows the mean moving average performance, alongside the choices of the winning model, throughout the course of the task by task order.

#### Mental health and social functioning

Children self-reported symptoms using the Strengths and Difficulties Questionnaire (SDQ)^29^ a widely used and well-validated measure of child and adolescent psychological functioning^30^. Mental health was assessed via the total difficulties score, comprising subscales on emotional symptoms, conduct problems, peer problems and hyperactivity/inattention. Social functioning was indexed via the five items on the prosocial subscale of the SDQ. For all items, participants select one of three responses in respect to themselves (not true = 0, somewhat true = 1, certainly true = 2). The total difficulties score comprising the four problem-focussed subscales (reflecting general vulnerability to mental health problems) ranges from 0 to 40. The prosocial behaviour score ranges from 0 to 10.

### Analysis

As a first step, a single mixed ANCOVA was carried out to explore the impact of within-participant (condition, valence) and between participant (gender and age) factors on overall learning rates. In order to test the hypotheses that there would be age-related increases in children’s adjustment of learning rates to match the volatility of the environment, we used the difference between learning rates in the stable (first) condition and volatile (second) condition (volatile learning rate -stable learning rate) as the outcome variable in a linear regression, with age (operationalised as a continuous variable, measured as the difference between date of birth and date of testing) as the predictor. We used the same regression model to test the hypothesis that there would be age-related differences in temperature. Finally, we carried out linear regression analyses to investigate whether learning rate paramaters/temperature was associated with mental health/social functioning scale scores. In all linear regressions, gender was included as an additional predictor variable. Because participants were recruited from 14 state-funded non fee-paying schools in London/the South East, where testing also took place, school was included as a random intercept in linear mixed models, using the R package lme4^31^.

## Results

**Fig. 3:**
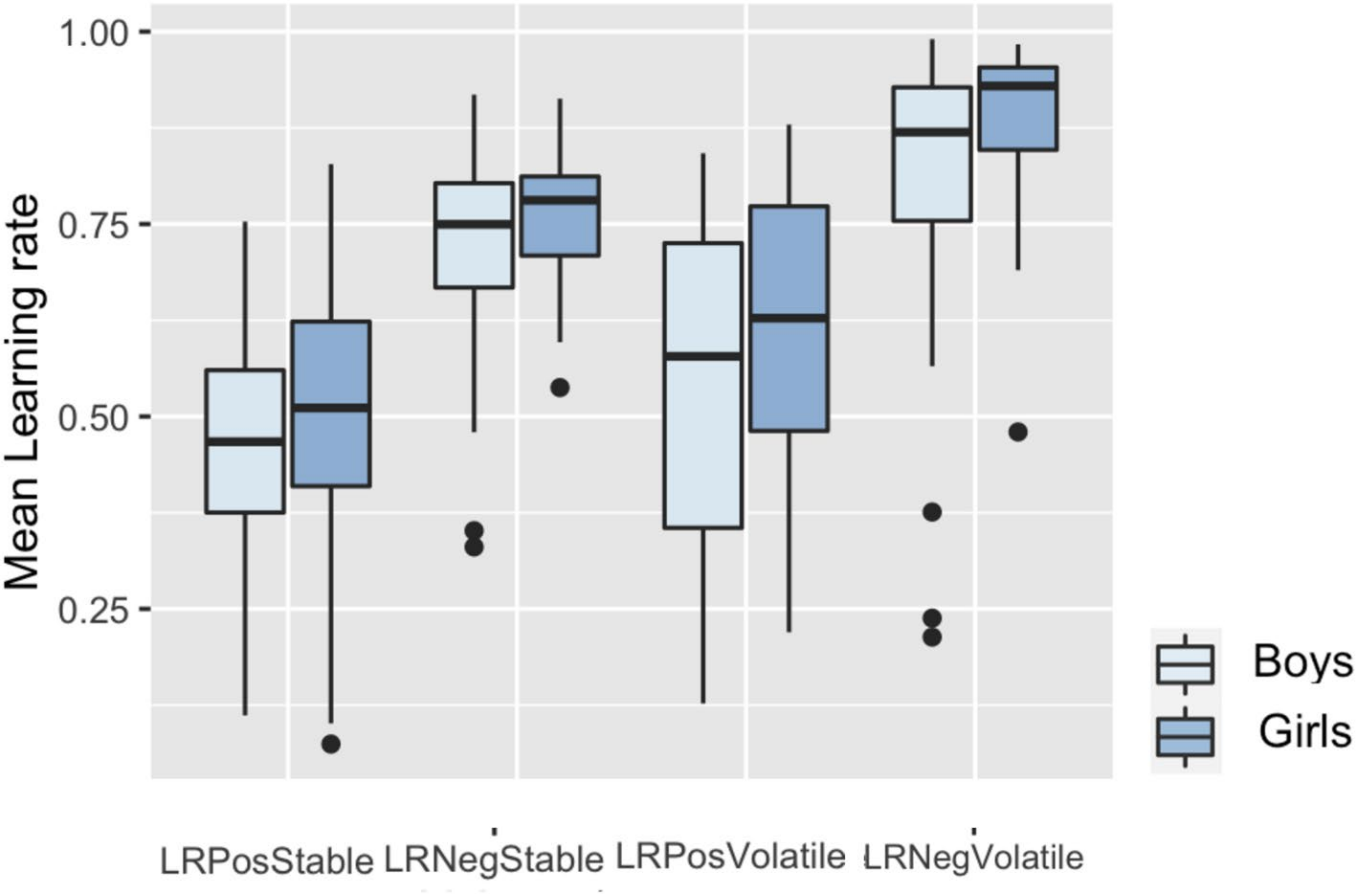
Learning Rates by Valence and Condition (order 1). Boxplots showing the IQR for positive stable, negative stable, positive volatile and negative volatile learning rates, with outliers represented by dots.

Among the 121 participants assigned to order 1 (stable followed by volatile conditions) a mixed ANCOVA with volatility condition (stable or volatile) and prediction error valence (positive or negative) as within-subjects factors male/female as between-subjects factors and age as a covariate showed that, as expected, learning rates were significantly greater in the volatile than the stable condition (main effect of volatility condition: *F*(1, 118) = 5.42, p = 0.02, ɳ_p_^2^ = 0.04; mean stable LR = 0.61, SD = 0.09; mean volatile LR = 0.71, SD = 0.15) and considerably greater following negative (i.e. omitted rewards) than positive outcomes (main effect of valence: *F*(1, 118) = 55.08, p < 0.001, ɳ_p_^2^ = 0.32; mean negative LR = 0.80, SD = 0.11; mean positive LR = 0.53, SD = 0.13). There was a significant effect of gender on learning rates (*F*(1, 118) = 5.28, p = 0.02, ɳ_p_^2^ = 0.04), but no interaction between gender and condition or valence. Girls had higher learning rates overall (mean = 0.69, SD = 0.08 than boys mean = 0.64, SD = 0.10), (see Fig. 3).

**Fig. 4:**
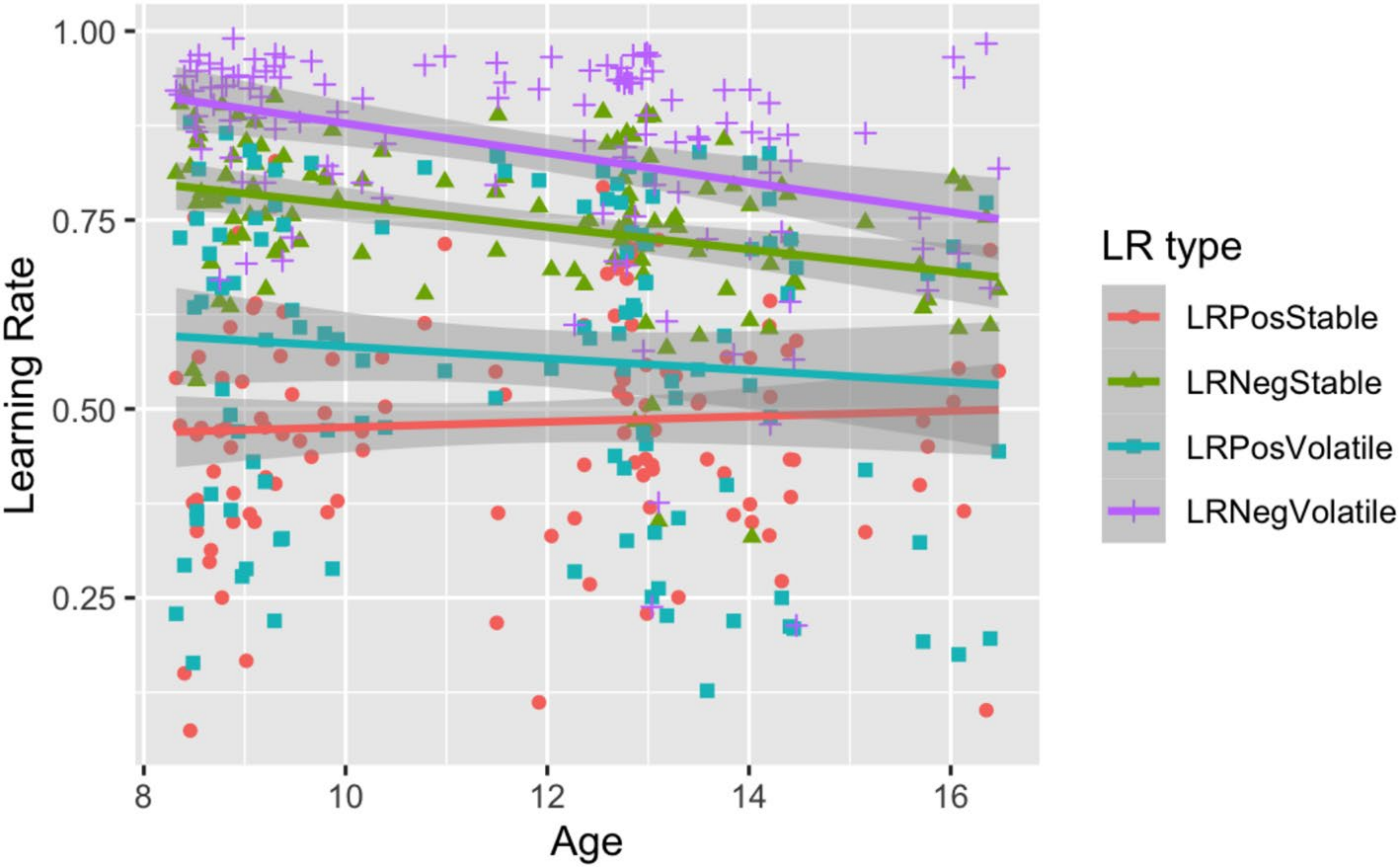
Plot showing the Relationship between Age and Learning Rates (order 1). The relationship between age (x-axis) and positive stable, negative stable, positive volatile and negative volatile learning rates (y-axis). The shaded regions indicate the standard errors.

There was a main effect of age on learning rates *F*(1, 118) = 4.49, p. = 0.04, ɳ_p_^2^ = 0.04), overall learning rates decreased with age, β = −0.24, p = 0.01. There was, however, a significant interaction between valence and age *F*(1, 118) = 9.78, p = 0.002, ɳ_p_^2^ = 0.08), revealing that learning rates decreased significantly by age only following negative (β = −0.39, p < 0.001) but not positive (β = −0.04, p = 0.66) outcomes (see Fig. 4). There was no interaction between age and condition.

When analysis was repeated to include data from participants assigned order 2 (volatile then stable phase,total n = 229) there was still a main effect of age; however the main effect of volatility was no longer significant (Supplementary Information S2). Contrary to expectations, when data from order 2 *only* was analysed, learning rates were significantly higher in the second, stable phase, than in the first, volatile phase (mean volatile LR = 0.63, SD = 0.15; stable LR = 0.72, SD = 0.10, t(107) = 6.08, p < 0.001).

### Adjustment in learning rates, by age

Adjustment in learning rates, was measured by the difference in learning rates between the stable (first) and volatile (second) conditions. This variable ranged from −0.25 to 0.47, M = 0.09, SD = 0.16. In a linear mixed effect model, age did not predict adjustment of learning rates (see Table 1). A similar result was found when the analysis was repeated including data from participants administered task order 2 (Supplementary Information S3).

**Table 1:** Age as a predictor of (a) Adjustment in learning rate and (b) Temperature (order 1).

	Adjustment in LR		Temperature					
Predictors	Estimate	CI	p	Estimate		CI		p
(intercept)	0.13	− 0.05–0.32	0.16	0.05		− 0.01–1.0		0.054
Gender	0.03	− 0.03–0.09	0.38	− 0.09		− 0.22–0.04		0.17
Age	− 0.01	− 0.02–0.01	0.30	0.04		0.00–0.08		0.03
Random effects								
σ^2^	0.03		0.10					
τ_00_ _School_	0.00		0.01					
N _School_	14		14					
Observations	121		121					
Marginal /Conditional R^2^	0.02/NA		0.102/0.186					

### Temperature by age

**Fig. 5:**
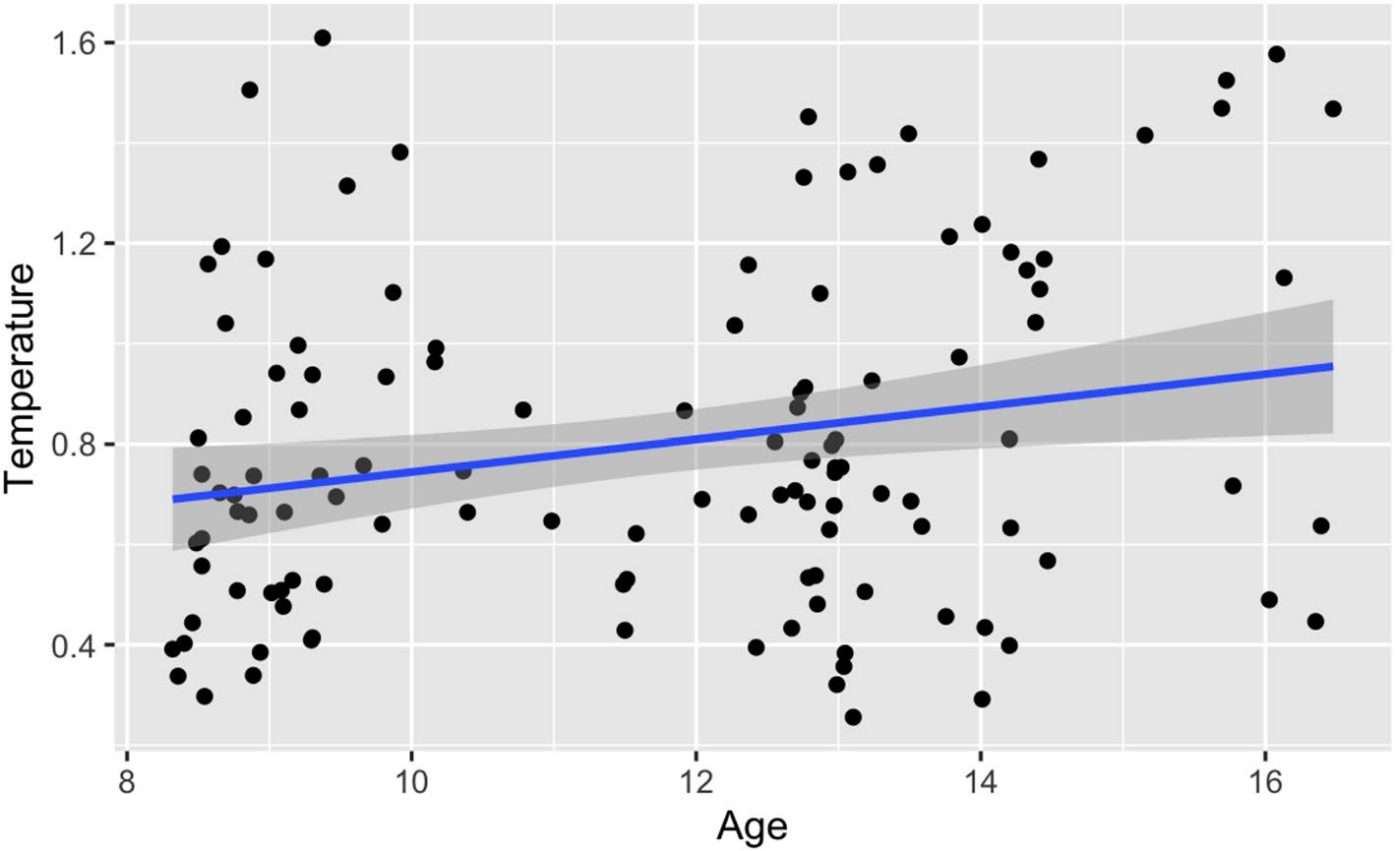
Plot showing the relationship between age and temperature (order 1). The relationship between age (x-axis) and temperature (y-axis). The shaded regions indicate standard errors.

Age was significantly associated with temperature scores (see Table 1). As shown in Fig. 5, as age increased, so did temperature, indicating an increase in exploratory behaviour with age. When including data from participants administered task order 2 also, there was a similar pattern of results although the relationship between temperature and age narrowly missed statistical significance (p = 0.08) (Supplementary Information S4).

### Associations with mental health and prosocial behaviour

The magnitudte of the increase in learning ates from stable to volatile conditions was not associated with overall mental health scores (total SDQ) (coefficient = 0.28, CI = −8.13–8.70, p = 0.95) or social functioning (prosocial SDQ) (coefficient = −0.55, CI = -2.89–1.78, p = 0.64). The same pattern of results was observed when data from children of all ages were included (8–16), (Supplementary Information S5) and when including data from participants administered task order 2 (Supplementary Information S6).

We further investigated whether any of the four individual learning rate parameters generated by the computational model were associated with either mental health (total SDQ) or prosocial behaviour scores in children aged eleven and above, adjusting the significance threshold to account for multiple comparisons: 0.05/8 = 0.006. There was one notable result: learning rates following positive outcomes in stable environments were associated with higher prosocial behaviour, which survived correction for multiple comparisons (Table 2). The same pattern of results was observed when including data from children of all ages (Supplementary Information S7) and when including data from participants administered task order 2 (Supplementary Information S8).

**Table 2:** Learning rates as predictors of mental health and prosocial behaviour.

	SDQ total scores			SDQ prosocial		
Predictors	Estimate	CI	p	Estimate	CI	p
(intercept)	21.72	− 1.48–44.92	0.07	3.9	− 2.15–9.94	0.21
Gender	2.32	− 1.16–5.79	0.19	0.2	− 0.70–1.11	0.66
Age	− 0.52	− 1.79–0.75	0.42	0	− 0.33–0.33	0.99
PosStable	0.34	− 9.79–10.48	0.95	4.13	1.49–6.77	0.002
PosVolatile	− 1.06	− 10.06–7.94	0.82	0.11	− 2.23–2.46	0.93
NegStable	− 9.01	− 23.41–5.39	0.22	0.37	− 3.38–4.12	0.85
NegVolatile	2.06	− 9.81–13.92	0.73	1.54	− 1.55–4.63	0.33
Random effects						
σ^2^		38.78		2.63		
τ_00_ _School_		0.00		0.00		
N _School_		9		9		
Observations		67		67		
Marginal R^2^ / Conditional R^2^		0.054 / NA		0.154/NA		

There was no significant association between temperature and either mental health (coefficient = -0.19, CI = −4.64–4.26, p = 0.93) or prosocial behaviour (coefficient = 0.31, CI = −0.93–1.55, p = 0.62). The same pattern of results was found when data from children of all ages were included (Supplementary Information S9) and when including data from participants administered task order 2 (Supplementary Information S10).

## Discussion

Learning from feedback in order to adjust behaviour appropriately appears to be fine-tuned between childhood and young adulthood, and operates atypically among individuals with mental health or social functioning difficulties. Utilising a computational approach, we did not find evidence that children’s adjustment of learning rates to suit the levels of uncertainty in the environment increases with age, nor that this adjustment is associated with better mental health and social functioning. Rather, we found that learning rates for worse-than-expected outcomes generally decrease with age, and that higher learning rates, specifically during positive stable environments, were associated with greater self-reported prosocial behaviour.

Our findings support several other studies that found a decrease in learning rates following negative outcomes as age of participants increases^1,2,3^ and are in keeping with developmental findings indicating that negative feedback has a bigger impact on learning in younger children^32^, and that children struggle to discern the true value of negative feedback^33^. The decline in negative learning rates through early adolesence may index increasing confidence in the stability of the environment acquired during this stage, in contrast to the relative developmental and environmental instability of the earlier years.

Following Behrens et al.^9^ and Manning et al.^23^ we found that learning rates increased between an initial stable, and second volatile condition. Findings on additional data from ‘order 2’ participants, who had higher learning rates in a secondary stable phase, following an initial volatile phase, cast doubt on whether the changes observed between conditions in the primary analysis reflect an adaptive adjustment to the environmental context. It has been suggested that flexibility in learning rates increases with age^7^ however our data suggests that one aspect of flexibility, the extent learning rates change in relation to the volatility in the environment, does not increase in the early adolescent period. The participants in this study demonstrated high learning rates in response to negative feedback, in stable and volatile conditions. More complex reward learning tasks or those with longer stable and volatile phases may be necessary to determine whether there are developmental changes in the modulation of learning rates to environmental uncertainty.

Our results are inconsistent with the hypothesis that increased adaptation in learning rates between stable and volatile periods acts as a protective factor against the development of mental health difficulties. However, we did find that children’s learning rates following better-than-expected outcomes in stable environments were positively associated with pro-social behaviour. Conversely, better-than-expected outcomes are less predictive in stable than in volatile environments^9^, meaning that this response is not necessarily adaptive. Simulating parameter settings to identify optimal performance in this task would be helpful to determine which of the behaviours observed were adaptive and how best to interpret this finding. Nevertheless, this association suggests that individuals who give weight to better than expected outcomes are more likely to show pro-social behaviour. This weighting may encourage individuals to take prosocial steps, such as social overtures, which may objectively be risky. Such steps could be important however, to promote the forging of social bonds which bring enjoyment, reduce loneliness and buffer the impact of developmental stressors.

The increase in exploratory behaviour with age observed in this study appears at odds with the majority of evidence documenting decreases in the temperature parameter with age, corresponding to greater selection of known rewarding choices. However, most of the existing evidence base compares adolescents and adults^2,6,17,34,35^,   whereas our study focuses on differences within primarily the early adolescent period^21^. Hormonal changes and concomitant structural and functional changes in the brain occur during this period^36,37^, affecting frontostriatal regions implicated in judgements of risk and reward^38^. Increases in exploratory behaviour shown here, could be construed as a form of risk taking, which has long been observed to increase in adolescence^39,40^.

An important limitation of our study is that it did not include very young children or young adults. Including a wider age range and a longitudinal framework would also be helpful in order to put the temperature findings in context: the increase in temperature from late childhood to early adolescence found here may be followed by age-related decreases in temperature into adulthood, as individuals reduce in risk taking. Although the task was kept short to maintain young children’s attention throughout, future studies could include longer or a larger number of stable/volatile blocks. The inclusion of punishment, as opposed to the absence of a positive reward here, would also be of interest.

In conclusion, we contribute to the growing evidence base that young children are particularly impacted by negative feedback and also provide evidence that temperature increases in the early adolescent period. Going forward, it will be important to understand the implications of this enhanced learning on children’s cognitions and behaviour, for example on their capacity to make rewarding decisions and regulate emotions. Our finding of an association between prosocial behaviour and greater learning from better-than-expected outcomes in stable environments warrants further investigation. It is possible, for example, that encouraging children to reflect on and incorporate unexpected positive outcomes into their decision making, may be helpful in promoting prosocial behaviour. Future work utilising a suite of different tasks and a longitudinal framework, as well as sensitive measures of mental health, social functioning and environmental contexts is needed to promote a better understanding of the role of reward learning in healthy socioemotional development.

## Supplementary Information

Supplementary Information.

## Data Availability

The participants of this study did not give written consent for their data to be shared publicly, so supporting data is not available. Queries regarding data accessibility should be directed to the corresponding author (EM).
