# Supplementary material for: Navigating a varying reward environment in childhood and adolescence
Source: Sci Rep. 2025 Jul 2;15:22715. doi: 10.1038/s41598-025-05725-3 (PMC12216166; doi:10.1038/s41598-025-05725-3)
Supplement: Supplementary file 1 — Supplementary Information. [file 41598_2025_5725_MOESM1_ESM.docx]

**Supplementary Information**

**S1 Fig 1: Model specification and comparison**


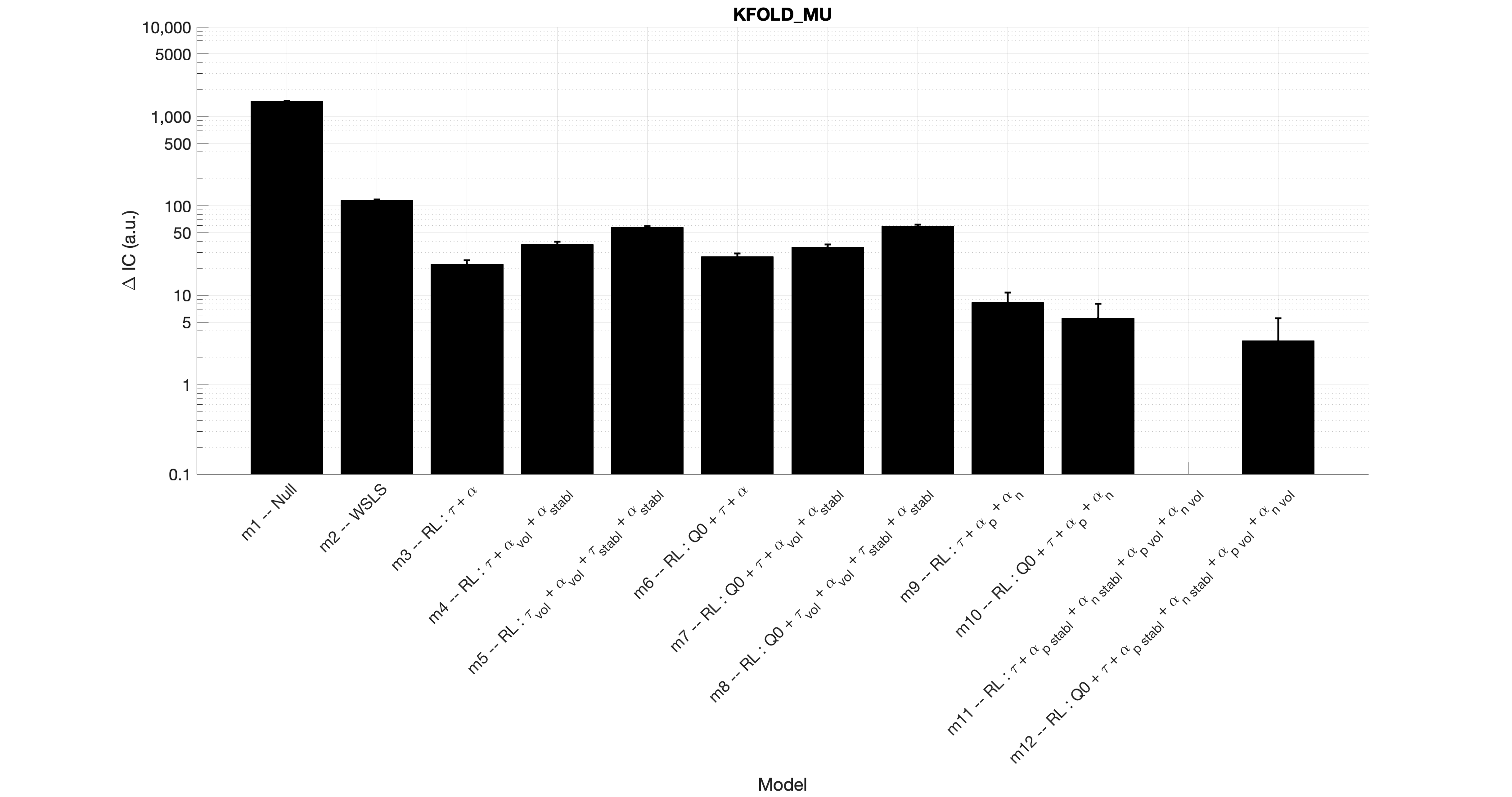


**S1: Model specification and comparison information**

The Widely Applicable Information Criterion (WAIC) scale and K-fold cross validation (K-fold CV) were used to compare model fits (Watanabe 2013; Vehtari et al., 2017)**.** The WAIC is akin to cross-validation, or other approximations such as the Akaike Information Criterion, but the WAIC is more sensitive, particularly in hierarchical modelling settings (Gelman et al., 2013). K-fold CV was used when diagnostic measures of the WAIC (pareto-k) suggested that the WAIC approximation was sub-optimal. While K-fold CV provides true cross validation metrics as opposed to the approximation yielded by the WAIC, it requires refitting each model K times with different sub-partitions of the data, and as such is substantially more computationally expensive to compute than WAIC. A lower K-fold CV or WAIC for each model indicates a stronger model evidence, and the relative difference between the winning model and another model can be used to establish the relative strength of the evidence for one model over another. This is akin to Bayes factors (Kass & Raftery 2012). We defined differences in model evidence (𝚫5-fold CV) as: weak (0-2); positive (2-6); strong (6-10); and very strong (>10) (Kass & Raftery, 1995).

For each model, two chains were produced with 1000 warm-up iterations and 4000 post warm-up iterations per chain. Model convergence was ensured through careful analysis of trace plots and monitoring of the Gelman-Rubin statistic (all potential scale reduction factors: R-hat < 1.1) (Brooks & Gelman 2012; Gelman & Rubin 1992). Recommendations for weakly informative priors in hierarchical logistic regression were used (Gelman & Hill 2007). Following recommendations (Gabry et al., 2017), prior predictive calibrations were performed on the winning model family to ensure that the prior choices led to adequate decision profiles.

In total twelve models were specified and estimated. All of the reinforcement learning models follow the same basic form, described here for Model 3 (Standard RL). The values of each stimulus (L = left, R = right) were initialised at 0.5 by default. On each trial the value of each stimulus is updated according to a standard prediction error rule: V(t+1) = V(t)+ α*δ(t), where t refers to the current trial, V represents the stimulus value, α represents the learning rate and δ represents the prediction error, i.e. how much better or worse is the outcome than expected. δ(t) = r(t)-V(t), where r represents the actual outcome received (1 for winning outcomes, 0 for non-winning outcomes). δ is scaled by the learning rate, α, a free parameter that is estimated separately for each participant. In order for the model to make choices, the stimulus values were entered into a softmax function (S1 Figure 5): P(L) = exp(τ*V(L))/(exp(V(L)/τ)+exp(V(R)/τ))), where P represents the probability that the model chooses a given stimulus (here, the left one), and τ is a free parameter representing the temperature or stochasticity of choices (lower values indicate more deterministic responding), which was also estimated separately for each participant.

As shown in the summary below, more complex models were then fit to allow for learning to be altered depending on either the valence of the outcome (win or no-win), and/or the volatile and stable environments: Model 4 had separate learning rates for stable and volatile environments; Model 5 had separate learning rates and separate temperature parameters for stable and volatile environments; Model 6 was the basic RL model (Model 3) with the addition of an initial bias parameter, Q, which allows the initial values to vary; Models 7 and 8 were similar to Models 4 and 5, respectively, with the addition of the initial bias parameter; Model 9 had separate learning rates for win and no-win outcomes; Model 10 was similar to Model 9, with the addition of the initial bias parameter; Model 11 (the winning model) had separate win and no-win learning rates, separately for the stable and volatile environments; and Model 12 was similar to Model 11 with the addition of the initial bias parameter. For all models, unless otherwise indicated, a single temperature parameter was used. In each model, all parameters were estimated separately for each participant.

- Model 1: Null model (random responding, serving as a benchmark)
- Model 2: Win-stay, Lose-Shift
- Model 3: Standard RL (1 temperature, 1 learning rate)
- Model 4: Standard RL with separate learning rates (1 temperature, 1 learning rate for stable environment 1 learning rate for volatile environment)
- Model 5: One standard RL model per environment, i.e. 1 temperature and 1 learning rate for each environment)
- Model 6: Model 3 + initial Q-value bias
- Model 7: Model 4 + initial Q-value bias
- Model 8: Model 5 + initial Q-value bias
- Model 9: Standard RL with separate learning rates for positive and negative outcomes (1 temperature, 1 learning rate for positive outcomes, 1 learning rate for negative outcomes)
- Model 10: Model 9 + initial Q-value bias
- **Model 11: Model 9 with separate learning rates for each environment (1 learning rate for positive outcomes in the stable environment, 1 learning rate for negative outcomes in the stable environment, 1 learning rate for positive outcomes in the volatile environment, 1 learning rate for negative outcomes in the volatile environment)**
- Model 12: Model 11 + initial Q-value bias

For all models, subject-level individual parameters (for the learning rates, and the initial Q-values) were beta distributed to enforce values between zero and one, while the temperature parameter was gamma distributed to enforce values between zero to infinity. Model parameters were estimated using Hamiltonian Markov Chain Monte Carlo (HMC) sampling.

**Hyperparameters for learning rates**

a_lr_pos_vol ~ normal(1.2, 5)

b_lr_pos_vol ~ normal(1.2, 5)

a_lr_pos_stabl ~ normal(1.2, 5)

b_lr_pos_stabl ~ normal(1.2, 5)

a_lr_neg_vol ~ normal(1.2, 5)

b_lr_neg_vol ~ normal(1.2, 5)

a_lr_neg_stabl ~ normal(1.2, 5)

b_lr_neg_stabl ~ normal(1.2, 5)

**Hyperparameters for temperature**

k_tau ~ normal(0.8, 20)

theta_tau ~ normal(1, 20)

**Weakly informative priors over parameters**

lr_pos_vol ~ beta(a_lr_pos_vol, b_lr_pos_vol)

lr_pos_stabl ~ beta(a_lr_pos_stabl, b_lr_pos_stabl)

lr_neg_vol ~ beta(a_lr_neg_vol, b_lr_neg_vol)

lr_neg_stabl ~ beta(a_lr_neg_stabl, b_lr_neg_stabl)

tau ~ gamma(k_tau ,theta_tau)

**S1 Fig 2: Participant data by model choices**


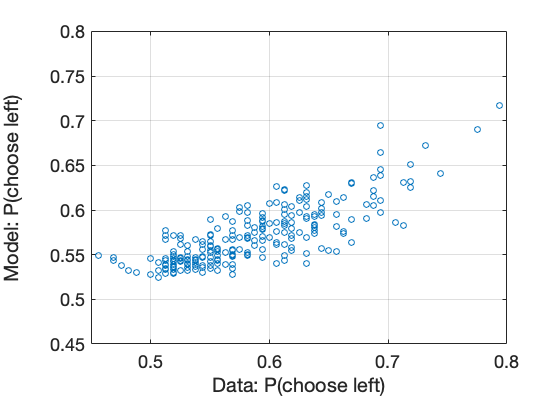


R^2:^ 0.647

***Note.*** In this plot each point represents a single individual, with their empirical probability of choosing the left stimulus overall on the x-axis, and the winning model’s predicted probability of choosing the left stimulus overall on the y-axis. To calculate the model-predicted values, 2,500 iterations were run per participant across 2 chains (5000 total), with the first 1000 iterations being discarded as warmup. The value used for the correlation was the mean value computed from averaging the chains. The correlation between empirical and modelled data is r2=0.647.

**S1 Fig 3: Parameter recovery: temperature**

**
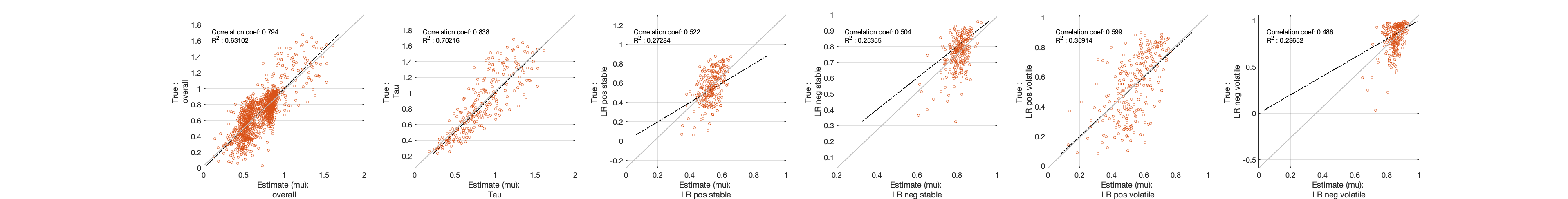
**

***Note.*** Each point represents a single individual with the estimated value of temperature on the x-axis and the true value on the y-axis. 2,500 iterations were run per participant across 2 chains (5000 total), with the first 1000 iterations being discarded as warmup. The parameter value used for the correlation was the mean value computed from averaging the chains. We defined the generating parameters to create synthetic data as the sets of parameters identified for each individual participant – i.e. the observed values for participants in the study.

**S1 Fig 4: Parameter recovery: learning rates**

**
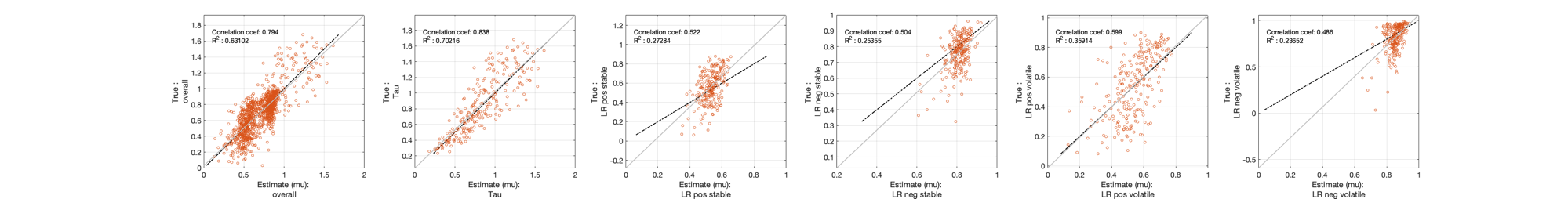
**

***Note.*** Plots showing the parameter recovery for individual learning rates (L-R: positive stable, negative stable, positive volatile and negative volatile). Each point represents a single individual with the estimated value of the learning rate on the x-axis and the true value on the y-axis. 2,500 iterations were run per participant across 2 chains (5000 total), with the first 1000 iterations being discarded as warmup. The parameter value used for the correlation was the mean value computed from averaging the chains.We defined the generating parameters to create synthetic data as the sets of parameters identified for each individual participant – i.e. the observed values for participants in the study.

**S1 Fig 5: Softmax function**

**S2: Learning rates by age, combined data from order 1 and order 2 (n = 229)**

When data from both orders was included, a mixed ANOVA with volatility condition (stable or volatile) and prediction error valence (positive or negative) as within-subjects factors and age, male/female and order as between-subjects factors did not reveal a significant main effect of volatility condition on learning rates (*F*(1,224) = 3.68, p = .06 ɳ_p_^2^ = .016). There was, however, a main effect of valence on learning rates (F (1, 224) = 76.53, p <.001, ɳ_p_^2^ = .26, with negative learning rates higher than positive learning rates. Mean negative LR = .79, SD = .11; mean positive LR = .54, SD = .13.

There was a significant main effect of age on learning rates, (F (1, 224) = 4.62, p = .03, ɳ_p_^2^ = .02, with learning rates reducing slightly as age increased, (r = -.16)

There was also a significant interaction between age and valence (F(1,224) = 10.63, p = .001, ɳ_p_^2^ = .045. Negative learning rates decreased significantly with age (r = -.28), but positive learning rates did not (r = -.02)

There was also a significant interaction between condition and order F(1, 224) = 70.22, p <.001, ɳ_p_^2^ = .24. In order 1 (stable first), learning rates were significantly higher in the volatile condition (M = .71, SD = .15) than in the stable condition (M = .61, SD = .09; t (120) = -6.31, p <.001). In order 2 (volatile first), however, learning rates were significantly higher in the stable condition (M = .72, SD = .10) than in the volatile condition (M = .63, SD = .15; t (107) = -6.08, p <.001.

There was also a significant three-way interaction between valence, condition and order. F (1,224) = 23.69, p <.001, ɳ_p_^2^ = .096.

**S3: Adjustment between stable and volatile conditions, combined data from order 1 and order 2**

In a mixed effects model with children's school as a random effect, and controlling for order, neither age (estimate = -.01, CI =-.02-.00, p =.08), nor gender (coefficient = .03, CI =-.01-.07, p = .11) predicted adjustment in learning rate between volatile and stable conditions.

**S4: Associations between age, gender and temperature, combined data from order 1 and order 2**

In a mixed effects model with children's school as a random effect, controlling for order, the association between age and temperature did not reach significance (coefficient = .03, CI = -.00-.06, p =.07), and nor did gender (coefficient = -.09, CI = -.19-.02, p =.10).

**S5: Associations with Mental Health and Prosocial Behaviour, order 1 only, including children of all ages.**

Among all children (aged 8-16) who were administered order 1, the difference in learning rates between stable and volatile conditions was not associated with children's mental health (total SDQ scores), (coefficient = 3.39, CI = -3.88-10.66, p =.36) or with their prosocial behaviour scores (prosocial SDQ scores), (coefficient = -0.89, CI = -2.86-1.07, p =.37).

**S6: Associations with Mental Health and Prosocial Behaviour, combined data from both order 1 and order 2**

Among children aged 11+ from both order 1 and order 2, the difference in learning rates between stable and volatile conditions was not associated with children's mental health (total SDQ scores), (coefficient = 1.53, CI = -3.79-6.85, p =.57) or prosocial behaviour (prosocial SDQ scores), (coefficient = -0.60, CI = -2.12-0.93, p =.45).

This was the same when all children aged 8-16 years were included: The difference in learning rates between stable and volatile conditions was not associated with children's mental health (total SDQ scores) (coefficient = 1.07, CI = -3.31-5.45, p =.63) or prosocial behaviour (prosocial SDQ scores) (coefficient = -.05, CI =-1.79-0.80, p =.45).

**S7: Associations between individual learning rates and Mental Health and Prosocial Behaviour, order 1 only, children of all ages.**

Using data from order 1 only, but this time including children aged 8-16, we again investigated whether any of the four individual learning rate parameters were associated with either children's mental health (total SDQ) or prosocial behaviour scores, with a revised alpha of .05/8 = .006. Again we only found one notable association. Children's self-reported prosocial behaviour was positively associated with children's positive, stable learning rates (coefficient = 2.11, CI = 0.00-4.23, p =.050)

**S8: Associations between individual learning rates and Mental Health and Prosocial Behaviour, combined data from order 1 and order 2.**

Using data from both order 1 and order 2 from children aged 11+, we also investigated whether any of the four individual learning rate parameters were associated with either children's mental health (total SDQ) or prosocial behaviour scores, with a revised alpha of .05/8 = .006. Again, there was only one notable result. Children's learning rates following positive outcomes in stable environments predicted greater levels of children's prosocial behaviour (coefficient = 2.08, CI = 0.25-3.92, p =.03)

Finally, we repeated the same analysis, again using data from both order 1 and order 2, but this time including all children aged 8-16. Once again, we found a positive association between children's prosocial behaviour and positive stable learning rates (coefficient = 1.49, CI = 0.04-2.94, p =.04). Here we also found a positive association between children's self-reported total difficulties scores and positive volatile learning rates (coefficient = 4.90, CI - 0.46-9.34, p =.03).

**S9: Associations between temperature and Mental Health and Prosocial Behaviour, order 1 only, children of all ages.**

Using data from order 1 only, but this time including children aged 8-16, we investigated whether temperature was associated with mental health or prosocial behaviour scores. We found no association between temperature and mental health (coefficient = -2.57, CI = - 6.27-1.13, p =.17), nor between temperature and prosocial behaviour (coefficient = 0.54, CI = -0.45-1.54, p =.29).

**S10: Associations between temperature and Mental Health and Prosocial Behaviour, combined data from order 1 and order 2.**

Using data from both order 1 and order 2 from children aged 11+ only, we did not find a significant association between temperature and mental health (coefficient = 0.78, CI = -1.88- 3.44, p =.57) nor between temperature and prosocial behaviour (coefficient = 0.05, CI = -0.73-0.82, p =.91).

Using data from both order 1 and order 2 from children aged 8-16, we did not find a significant association between temperature and mental health (coefficient = -1.03, CI = -3.19-1.13, p =.35), nor between temperature and prosocial behaviour (coefficient = 0.19, CI = -0.45-0.93, p =.56).

**S11: Task performance data**

Table S11 below shows that, on average, participants chose the rewarded pirate 65% of the time in the stable condition, and 62% of time in the volatile condition. This difference was significant, (t(120) = 3.1, p = .002), revealing that performance was significantly better in the stable condition.

**Table S11**

Proportion of trials participants chose the pirate with the higher reward probability (stable first, n = 121).

| **Stable Condition** | | **Volatile Condition** | | | | |
| --- | --- | --- | --- | --- | --- | --- |
| **Trials** | **Trials 1-80** | **Trials 81-100** | **Trials 101-120** | **Trials 121-140** | **Trials**  **141-160** | **All**  **81-160** |
| **Rewarded**  **pirate** | **Red (75:25)** | **Blue (80:20)** | **Red (80:20)** | **Blue (80:20)** | **Red**  **(80:20)** |  |
| Mean (SD) % | .65 (.08) | .57(.12) | .62 (.13) | .59 (.14) | .70 (.14) | .62 (.08) |
| Min-Max | .51 - .89 | .3 - .9 | .3 -.9 | .15- 9 | .05-1.0 | .42 - .81 |

There was no significant association between age and percent correct in the stable phase (r = .007, p =.94); however there was a small, but significant, association between age and percent correct in the volatile stage (r = -.23, p =.01), indicating that younger participants were more likely to select the most rewarded pirate in the volatile condition. The relationships are shown in the scatterplot below (Figure S11a). Although it may seem surprising that younger participants performed better, we note that younger participants also tended to have higher negative learning rates. As discussed in more detail below (and shown in Figure S11c), higher negative learning rates were associated with better performance in the volatile condition; this is expected, as higher learning rates reflect a greater weighting of recent outcomes on value estimates, and therefore are advantageous in volatile conditions (Behrens, 2007).

**Figure S11a**


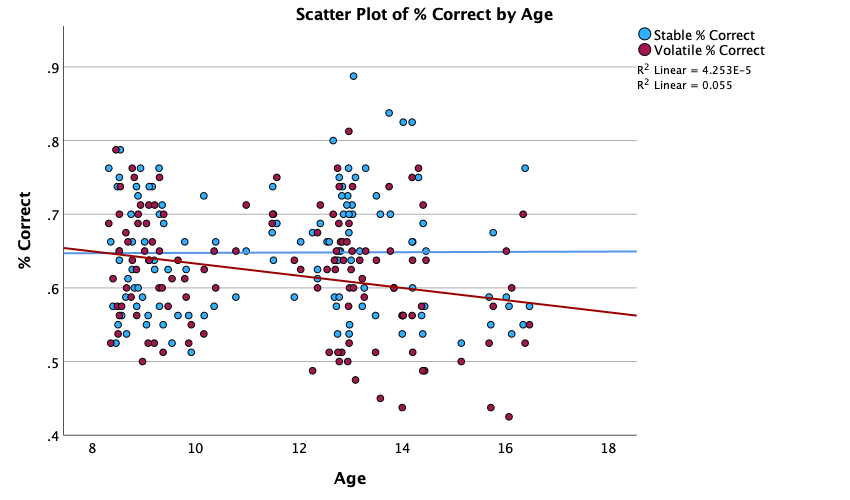


*Note.* The relationship between participant age (x-axis) and the mean proportion of times a participant chose the most rewarded pirate, ( y-axis), in stable and volatile conditions.

Figure S11b shows that in the stable condition, there was a small, but significant, positive association between learning rates for positive (better-than expected) outcomes and performance (r = .196, p = .03). In contrast there was a small, but significant, negative association between learning rates for negative (poorer-than-expected) outcomes and performance (r = -.202, p =.03). The association with negative learning rates is consistent with Behrens and colleagues’ (2007) proposal that lower learning rates are advantageous in stable conditions. However, higher positive learning rates were also apparently advantageous for performance, perhaps reflecting the uncertainty inherent in the initial trials, and the fact that without punishments as such (coins were not lost following a 'wrong' choice), rewards may have been more salient.

In the volatile condition, the relationships between learning rates and task performance were clearer (Figure S11c): there were significant positive associations between learning rates and task performance for both positive (r = .50, p <.001) and negative outcomes (r = .39, p <.001). This finding is again consistent with the proposal that high learning rates are advantageous in volatile conditions, where recent outcomes are more predictive than past ones. These stronger correlations may also reflect the ratio of 80:20 in the stable condition vs 75:25 in the volatile condition, perhaps making unexpected outcomes more salient to participants, particularly at the end of the volatile condition by which time the contingencies had switched several times.

**Figure S11b**


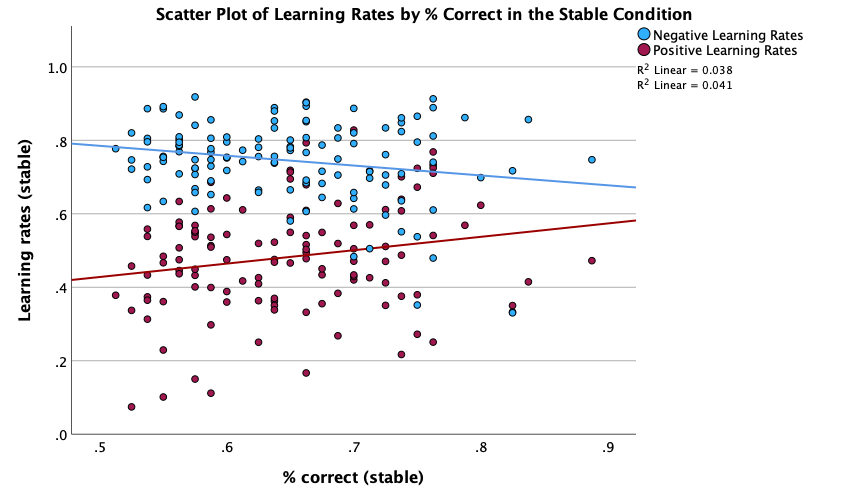


*Note.* The relationship between the mean proportion of times participants chose the most rewarded pirate (x-axis) and their mean learning rates (y-axis), for negative and positive learning rates, within the stable condition.

**Figure S11c**


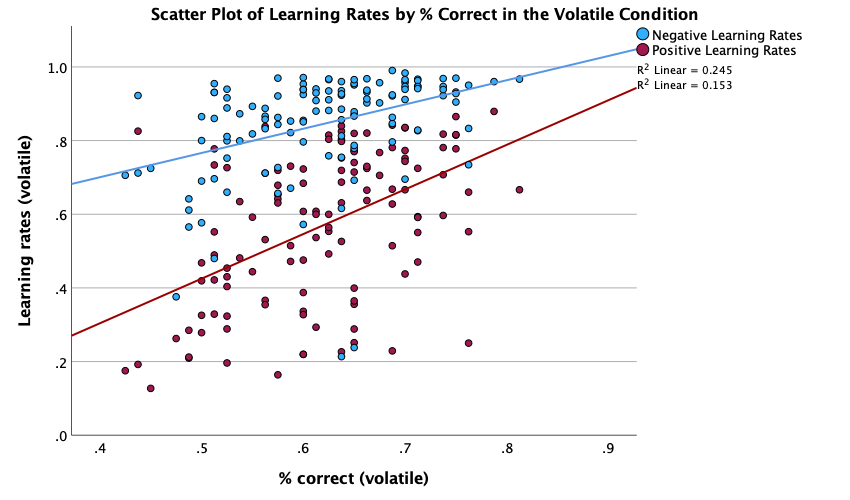


*Note.* The relationship between the mean proportion of times participants chose the most rewarded pirate (x-axis) and their mean learning rates (y-axis), for negative and positive learning rates, within the volatile condition.

Finally, we also investigated the relationship between the temperature parameter and task performance. As shown in Figure S11d (below), temperature was negatively associated with performance in both stable and volatile conditions, indicating that exploratory behaviour was disadvantageous in this task. This is in keeping with the findings that exploratory behaviour increased with age, whereas the likelihood of selecting the most rewarding pirate decreased with age, at least in the volatile stage.

**Figure S11d**


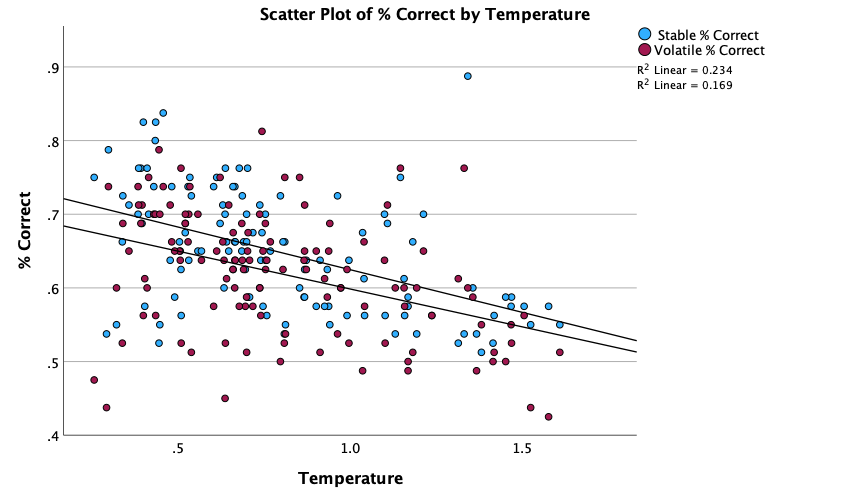


*Note.* The relationship between participants' temperature scores (x-axis) and the mean proportion of times the most rewarded pirate was chosen (y-axis), in stable and volatile conditions.
